# Supplementary material for: A Likelihood Approach for Real-Time Calibration of Stochastic Compartmental Epidemic Models
Source: PLoS Comput Biol. 2017 Jan 17;13(1):e1005257. doi: 10.1371/journal.pcbi.1005257 (PMC5240920; doi:10.1371/journal.pcbi.1005257)
Supplement: S1 File — (TAR.GZ) [file pcbi.1005257.s014.tar.gz › HSPH_Online-SI-Revision/IPoi09/n10-mild/n10-mild_BENCH2_table-8.pdf]

1	newly 3 weeks spec	108.	203.273	{38., 433.}	119.318	0.390268	newly 3 weeks cumul	274.	471.068	{83., 1050.}	107.191	0.37605	newly 5 weeks spec	177.	316.477	{86., 538.}	105.277	0.360592	newly 5 weeks cumul	569.	1048.02	{233., 2100.}	113.505	0.377742	newly 58 weeks cumul	3351.	6780.93	{4941., 7903.}	106.864	0.351156
2	newly 3 weeks spec	133.	373.545	{46., 801.}	205.65	0.468243	newly 3 weeks cumul	288.	716.848	{92., 1608.}	178.714	0.437574	newly 5 weeks spec	307.	738.5	{226., 1143.}	154.215	0.400617	newly 5 weeks cumul	831.	2010.58	{460., 3667.}	161.427	0.409101	newly 63 weeks cumul	4314.	8464.98	{7221., 9315.}	96.2213	0.289378
3	newly 3 weeks spec	119.	1183.67	{182., 1890.}	912.456	1.17029	newly 3 weeks cumul	252.	3309.53	{256., 5099.}	1231.08	1.3075	newly 5 weeks spec	232.	469.222	{119., 1076.}	134.128	0.603889	newly 5 weeks cumul	651.	4583.49	{2067., 7100.}	621.847	1.13137	newly 66 weeks cumul	4852.	5060.38	{2142., 7568.}	29.5709	0.500772
4	newly 3 weeks spec	134.	442.556	{101., 783.}	248.905	0.630232	newly 3 weeks cumul	346.	1012.31	{179., 2025.}	214.464	0.596027	newly 5 weeks spec	273.	570.6	{177., 896.}	127.163	0.507182	newly 5 weeks cumul	805.	2100.18	{494., 3324.}	180.182	0.570567	newly 61 weeks cumul	4621.	5472.93	{3397., 8427.}	37.7557	0.276547
5	newly 3 weeks spec	76.	203.056	{98., 296.}	175.877	0.490004	newly 3 weeks cumul	221.	605.556	{193., 885.}	183.761	0.472704	newly 5 weeks spec	121.	194.	{93., 264.}	73.6455	0.314954	newly 5 weeks cumul	442.	997.75	{375., 1360.}	135.904	0.409449	newly 41 weeks cumul	2815.	3050.14	{1736., 4791.}	27.8261	0.177745
6	newly 3 weeks spec	89.	1328.83	{57., 2340.}	1412.92	1.31347	newly 3 weeks cumul	165.	2458.09	{97., 4975.}	1409.78	1.29491	newly 5 weeks spec	215.	1235.2	{316., 2202.}	493.032	0.990785	newly 5 weeks cumul	499.	5168.48	{551., 7726.}	954.279	1.25579	newly 67 weeks cumul	3503.	6933.13	{3730., 9197.}	116.437	0.698463
7	newly 3 weeks spec	131.	679.231	{62., 1051.}	435.173	0.842366	newly 3 weeks cumul	306.	1708.23	{99., 2834.}	475.591	0.833587	newly 5 weeks spec	265.	662.949	{188., 1151.}	165.689	0.56094	newly 5 weeks cumul	790.	3086.08	{645., 4629.}	305.586	0.728025	newly 63 weeks cumul	3676.	4980.82	{2860., 7781.}	48.1697	0.334725
8	newly 3 weeks spec	120.	276.05	{153., 380.}	130.042	0.339856	newly 3 weeks cumul	269.	897.8	{451., 1199.}	233.755	0.499861	newly 5 weeks spec	198.	213.55	{87., 345.}	38.5101	0.163234	newly 5 weeks cumul	652.	1356.6	{762., 1918.}	108.067	0.301394	newly 58 weeks cumul	4528.	2214.95	{1354., 2992.}	51.0833	0.33366
9	newly 3 weeks spec	122.	308.03	{35., 575.}	179.732	0.489091	newly 3 weeks cumul	305.	612.182	{74., 1150.}	134.138	0.442221	newly 5 weeks spec	202.	604.273	{128., 970.}	215.047	0.526725	newly 5 weeks cumul	662.	1666.92	{267., 2895.}	173.348	0.483269	newly 62 weeks cumul	4588.	8269.17	{7171., 9150.}	83.265	0.326724
10	newly 3 weeks spec	146.	347.625	{110., 601.}	147.175	0.364465	newly 3 weeks cumul	372.	1229.81	{484., 1779.}	235.837	0.500551	newly 5 weeks spec	191.	210.813	{83., 472.}	52.2579	0.258975	newly 5 weeks cumul	713.	1709.06	{590., 2539.}	145.1	0.368078	newly 59 weeks cumul	3268.	2326.	{1341., 3760.}	33.786	0.210497
11	newly 3 weeks spec	101.	786.346	{394., 1094.}	678.561	0.854108	newly 3 weeks cumul	225.	2172.23	{993., 2972.}	865.436	0.955639	newly 5 weeks spec	200.	506.731	{131., 783.}	170.173	0.428947	newly 5 weeks cumul	597.	3376.54	{2471., 4212.}	465.584	0.741352	newly 64 weeks cumul	2927.	4130.62	{3139., 5323.}	46.9055	0.167204
12	newly 3 weeks spec	49.	604.061	{102., 1148.}	1137.35	0.987221	newly 3 weeks cumul	71.	950.621	{139., 2041.}	1243.17	1.01153	newly 5 weeks spec	277.	1406.08	{627., 1844.}	411.733	0.727003	newly 5 weeks cumul	466.	3437.85	{1068., 5273.}	641.858	0.859327	newly 66 weeks cumul	3893.	8316.02	{6497., 9283.}	116.645	0.396195
13	newly 3 weeks spec	133.	213.792	{53., 410.}	90.3822	0.334593	newly 3 weeks cumul	290.	570.292	{168., 1269.}	119.353	0.334258	newly 5 weeks spec	295.	263.542	{76., 452.}	41.4831	0.24725	newly 5 weeks cumul	831.	1070.79	{327., 1811.}	64.3753	0.26873	newly 58 weeks cumul	4651.	4031.08	{3019., 5091.}	21.5223	0.114072
14	newly 3 weeks spec	178.	452.563	{206., 637.}	162.992	0.408937	newly 3 weeks cumul	407.	1199.19	{463., 1723.}	204.023	0.466907	newly 5 weeks spec	248.	485.188	{291., 701.}	103.881	0.307541	newly 5 weeks cumul	867.	2172.06	{1056., 3046.}	156.985	0.400684	newly 59 weeks cumul	4541.	4889.47	{3204., 6926.}	27.7038	0.117568
15	newly 3 weeks spec	89.	483.892	{141., 752.}	454.084	0.785827	newly 3 weeks cumul	247.	1303.86	{286., 1919.}	438.866	0.780613	newly 5 weeks spec	149.	477.162	{233., 835.}	229.675	0.559389	newly 5 weeks cumul	522.	2268.89	{790., 3484.}	344.76	0.718696	newly 61 weeks cumul	4384.	4725.68	{2690., 7404.}	37.627	0.266485
16	newly 3 weeks spec	118.	333.154	{0., 601.}	227.901	0.892773	newly 3 weeks cumul	242.	833.462	{6., 1623.}	287.54	0.880689	newly 5 weeks spec	266.	310.538	{0., 531.}	65.2111	0.822294	newly 5 weeks cumul	698.	1476.35	{6., 2486.}	156.453	0.820237	newly 62 weeks cumul	4382.	2662.38	{6., 3635.}	40.2679	0.745419
17	newly 3 weeks spec	32.	39.7872	{5., 99.}	88.9628	0.432468	newly 3 weeks cumul	75.	96.2128	{13., 226.}	90.2411	0.385901	newly 5 weeks spec	45.	69.2128	{8., 171.}	106.383	0.406603	newly 5 weeks cumul	154.	218.787	{33., 504.}	97.9414	0.382693	newly 59 weeks cumul	3428.	6451.15	{4527., 7610.}	92.6525	0.317371
18	newly 3 weeks spec	96.	220.833	{61., 406.}	141.377	0.341983	newly 3 weeks cumul	238.	744.111	{312., 1105.}	214.799	0.45684	newly 5 weeks spec	159.	165.5	{15., 393.}	79.9092	0.481625	newly 5 weeks cumul	518.	1111.28	{348., 2058.}	125.558	0.331929	newly 59 weeks cumul	2823.	1790.61	{348., 3784.}	47.0953	0.356023
19	newly 3 weeks spec	250.	617.	{358., 881.}	146.8	0.36969	newly 3 weeks cumul	538.	1786.8	{1273., 2434.}	232.119	0.503718	newly 5 weeks spec	410.	433.16	{122., 627.}	45.2585	0.210033	newly 5 weeks cumul	1241.	2773.08	{1865., 3475.}	123.455	0.337825	newly 62 weeks cumul	4288.	3622.92	{2402., 5048.}	21.1521	0.111509
20	newly 3 weeks spec	215.	188.273	{13., 415.}	59.8732	0.59467	newly 3 weeks cumul	419.	503.727	{165., 868.}	68.1276	0.534652	newly 5 weeks spec	303.	162.818	{0., 329.}	52.4452	0.860993	newly 5 weeks cumul	946.	838.909	{166., 1421.}	47.0306	0.561097	newly 63 weeks cumul	4612.	1327.73	{166., 2316.}	71.2115	0.961551
21	newly 3 weeks spec	171.	347.357	{34., 703.}	139.515	0.495788	newly 3 weeks cumul	333.	697.375	{70., 1537.}	148.751	0.52227	newly 5 weeks spec	270.	659.393	{140., 1097.}	163.638	0.515909	newly 5 weeks cumul	859.	1872.84	{301., 3385.}	145.125	0.513767	newly 63 weeks cumul	4235.	7429.27	{5181., 9257.}	83.4968	0.410138
22	newly 3 weeks spec	162.	295.674	{32., 510.}	110.996	0.387294	newly 3 weeks cumul	330.	747.605	{110., 1379.}	153.467	0.43089	newly 5 weeks spec	235.	382.721	{96., 599.}	83.523	0.296407	newly 5 weeks cumul	771.	1469.72	{198., 2326.}	114.303	0.370222	newly 59 weeks cumul	3786.	5893.21	{3021., 8256.}	63.873	0.20549
23	newly 3 weeks spec	145.	119.348	{25., 249.}	61.4693	0.569399	newly 3 weeks cumul	243.	321.261	{63., 733.}	84.5947	0.346823	newly 5 weeks spec	283.	178.174	{0., 481.}	67.2454	0.780317	newly 5 weeks cumul	754.	646.435	{166., 1467.}	60.0853	0.396222	newly 64 weeks cumul	3569.	3780.87	{252., 8285.}	77.2656	0.524166
24	newly 3 weeks spec	208.	421.904	{80., 699.}	131.759	0.43972	newly 3 weeks cumul	535.	989.058	{179., 1731.}	119.723	0.413291	newly 5 weeks spec	227.	590.385	{184., 850.}	172.009	0.470487	newly 5 weeks cumul	960.	2096.12	{451., 3292.}	140.453	0.421412	newly 60 weeks cumul	2818.	7301.29	{6550., 8452.}	163.03	0.460153
25	newly 3 weeks spec	8.	68.6984	{0., 215.}	794.048	0.885291	newly 3 weeks cumul	23.	106.556	{2., 314.}	399.31	0.636561	newly 5 weeks spec	113.	300.127	{0., 919.}	235.497	0.861326	newly 5 weeks cumul	209.	556.524	{16., 1905.}	232.43	0.717915	newly 67 weeks cumul	3078.	7394.7	{20., 9264.}	165.587	0.80032
26	newly 3 weeks spec	173.	362.692	{169., 560.}	113.117	0.299898	newly 3 weeks cumul	463.	1259.62	{716., 1714.}	173.999	0.418924	newly 5 weeks spec	234.	235.692	{57., 503.}	54.3393	0.269017	newly 5 weeks cumul	924.	1781.08	{1007., 2404.}	95.7043	0.274334	newly 58 weeks cumul	3097.	2446.58	{1135., 3865.}	35.1345	0.198241
27	newly 3 weeks spec	144.	294.409	{141., 377.}	113.289	0.327664	newly 3 weeks cumul	310.	811.591	{344., 1070.}	167.199	0.408954	newly 5 weeks spec	330.	273.864	{149., 350.}	23.4573	0.129692	newly 5 weeks cumul	867.	1380.77	{852., 1691.}	67.8043	0.229179	newly 60 weeks cumul	4073.	2700.36	{1910., 3384.}	33.7009	0.187661
28	newly 3 weeks spec	96.	167.5	{60., 271.}	89.5182	0.279428	newly 3 weeks cumul	277.	535.188	{240., 829.}	102.956	0.29901	newly 5 weeks spec	113.	148.406	{66., 262.}	62.1958	0.237847	newly 5 weeks cumul	500.	843.719	{370., 1321.}	81.3063	0.253264	newly 38 weeks cumul	2478.	2133.53	{802., 3967.}	49.86	0.290779
29	newly 3 weeks spec	215.	317.426	{91., 637.}	86.1585	0.304841	newly 3 weeks cumul	511.	662.444	{161., 1443.}	79.7855	0.326932	newly 5 weeks spec	312.	540.63	{240., 907.}	86.5859	0.27074	newly 5 weeks cumul	1054.	1633.17	{537., 3038.}	83.6654	0.284298	newly 61 weeks cumul	4420.	7798.46	{6558., 8592.}	76.6646	0.243199
30	newly 3 weeks spec	272.	263.818	{40., 448.}	49.6324	0.315099	newly 3 weeks cumul	607.	988.727	{179., 1464.}	89.5462	0.316376	newly 5 weeks spec	287.	152.636	{25., 351.}	54.7355	0.577066	newly 5 weeks cumul	1151.	1333.18	{238., 2142.}	55.904	0.276051	newly 60 weeks cumul	4309.	1887.09	{387., 3219.}	56.2058	0.463336
31	newly 3 weeks spec	58.	866.944	{50., 1798.}	1413.55	1.22286	newly 3 weeks cumul	93.	1270.86	{79., 2674.}	1285.6	1.18206	newly 5 weeks spec	156.	1637.42	{475., 2202.}	966.293	1.21602	newly 5 weeks cumul	351.	4386.67	{723., 6680.}	1166.42	1.27939	newly 67 weeks cumul	3019.	7752.07	{4893., 9269.}	173.442	0.765374
32	newly 3 weeks spec	113.	255.147	{135., 356.}	127.564	0.332143	newly 3 weeks cumul	325.	780.588	{442., 1086.}	140.615	0.356749	newly 5 weeks spec	137.	223.471	{105., 338.}	75.1825	0.233332	newly 5 weeks cumul	581.	1242.38	{728., 1668.}	115.121	0.308825	newly 40 weeks cumul	3452.	3089.97	{1228., 5538.}	38.6349	0.20711
33	newly 3 weeks spec	152.	247.625	{23., 376.}	88.4046	0.429141	newly 3 weeks cumul	297.	659.708	{53., 1193.}	144.571	0.457029	newly 5 weeks spec	297.	263.458	{41., 375.}	29.0825	0.345519	newly 5 weeks cumul	807.	1186.46	{123., 1821.}	70.2757	0.345029	newly 61 weeks cumul	4655.	2751.29	{2258., 3386.}	40.896	0.37186
34	newly 3 weeks spec	162.	381.72	{73., 608.}	157.16	0.625805	newly 3 weeks cumul	346.	1115.28	{224., 1828.}	240.532	0.647158	newly 5 weeks spec	262.	309.4	{77., 583.}	53.8168	0.445779	newly 5 weeks cumul	775.	1778.12	{401., 2693.}	149.368	0.570932	newly 61 weeks cumul	4348.	3078.	{1904., 4398.}	30.3073	0.396857
35	newly 3 weeks spec	234.	556.8	{259., 889.}	146.923	0.449114	newly 3 weeks cumul	519.	1501.68	{652., 2243.}	197.404	0.496698	newly 5 weeks spec	339.	566.2	{241., 912.}	85.944	0.351546	newly 5 weeks cumul	1155.	2656.88	{1308., 4013.}	137.188	0.413758	newly 61 weeks cumul	4450.	4992.98	{2273., 7713.}	37.823	0.225954
36	newly 3 weeks spec	156.	496.	{99., 955.}	232.525	0.474278	newly 3 weeks cumul	432.	967.923	{151., 2023.}	148.251	0.380091	newly 5 weeks spec	336.	876.6	{355., 1199.}	166.9	0.412412	newly 5 weeks cumul	978.	2549.98	{687., 4187.}	172.168	0.410802	newly 63 weeks cumul	4342.	8394.8	{7221., 9157.}	93.3395	0.283835
37	newly 3 weeks spec	100.	656.828	{0., 1187.}	591.31	1.21937	newly 3 weeks cumul	185.	1518.34	{0., 2803.}	755.098	1.28	newly 5 weeks spec	184.	440.448	{0., 941.}	179.629	0.916759	newly 5 weeks cumul	548.	2529.59	{0., 3931.}	396.048	1.19926	newly 66 weeks cumul	3080.	3157.55	{0., 4413.}	37.9568	0.800856
38	newly 3 weeks spec	166.	525.	{40., 818.}	234.596	0.637096	newly 3 weeks cumul	393.	1505.82	{103., 2266.}	300.881	0.714885	newly 5 weeks spec	256.	435.179	{79., 790.}	99.4838	0.427585	newly 5 weeks cumul	843.	2423.07	{227., 3479.}	205.186	0.626524	newly 62 weeks cumul	4770.	4093.75	{2428., 5558.}	25.0022	0.273612
39	newly 3 weeks spec	121.	397.385	{169., 622.}	228.417	0.47549	newly 3 weeks cumul	299.	1396.15	{879., 1772.}	366.941	0.638001	newly 5 weeks spec	257.	185.538	{28., 456.}	52.4693	0.377968	newly 5 weeks cumul	736.	1860.54	{1049., 2416.}	152.791	0.38525	newly 63 weeks cumul	3570.	2248.38	{1500., 2887.}	37.5242	0.222613
40	newly 3 weeks spec	88.	193.387	{126., 239.}	122.617	0.333271	newly 3 weeks cumul	286.	585.	{406., 778.}	110.208	0.315421	newly 5 weeks spec	121.	190.065	{112., 244.}	61.7169	0.200916	newly 5 weeks cumul	519.	967.29	{662., 1218.}	90.9379	0.274657	newly 36 weeks cumul	2280.	3302.61	{1721., 4565.}	55.59	0.188072
41	newly 3 weeks spec	60.	1769.33	{99., 2816.}	2866.79	1.58049	newly 3 weeks cumul	169.	2891.7	{125., 4990.}	1629.54	1.33918	newly 5 weeks spec	165.	1314.97	{288., 2147.}	714.862	1.10469	newly 5 weeks cumul	429.	6146.3	{2224., 8199.}	1350.53	1.35265	newly 67 weeks cumul	3396.	7362.49	{4830., 9194.}	134.699	0.690142
42	newly 3 weeks spec	170.	70.0714	{0., 237.}	91.6387	1.4976	newly 3 weeks cumul	363.	333.143	{21., 824.}	92.4833	0.595405	newly 5 weeks spec	211.	32.5714	{0., 204.}	84.631	2.39485	newly 5 weeks cumul	769.	413.	{21., 1252.}	86.9404	0.817248	newly 59 weeks cumul	3469.	477.214	{21., 1639.}	86.2435	1.33882
43	newly 3 weeks spec	65.	115.231	{0., 386.}	151.834	1.10219	newly 3 weeks cumul	145.	333.769	{13., 991.}	182.175	0.690595	newly 5 weeks spec	321.	132.077	{0., 415.}	68.9192	1.37606	newly 5 weeks cumul	595.	594.385	{13., 1473.}	70.7951	0.723812	newly 65 weeks cumul	3665.	1162.08	{13., 2411.}	68.2926	1.11815
44	newly 3 weeks spec	156.	301.31	{53., 666.}	124.757	0.450599	newly 3 weeks cumul	333.	839.	{103., 1728.}	181.526	0.541101	newly 5 weeks spec	232.	290.759	{51., 560.}	58.5315	0.332152	newly 5 weeks cumul	768.	1433.	{209., 2753.}	115.324	0.465288	newly 61 weeks cumul	4274.	3160.62	{1872., 4414.}	28.606	0.296788
45	newly 3 weeks spec	48.	10.74	{0., 24.}	97.25	1.8449	newly 3 weeks cumul	71.	17.16	{0., 36.}	94.5352	1.64797	newly 5 weeks spec	143.	59.13	{0., 120.}	109.769	2.12855	newly 5 weeks cumul	285.	101.57	{0., 204.}	103.667	1.95714	newly 68 weeks cumul	4420.	3412.51	{0., 9026.}	91.6817	2.34504
46	newly 3 weeks spec	89.	1040.71	{409., 1530.}	1070.5	1.01958	newly 3 weeks cumul	211.	1889.17	{602., 3120.}	797.107	0.891817	newly 5 weeks spec	199.	1433.43	{889., 1763.}	620.317	0.837241	newly 5 weeks cumul	585.	4730.03	{2815., 6080.}	708.553	0.88542	newly 65 weeks cumul	3593.	8151.86	{6302., 9218.}	126.882	0.349503
47	newly 3 weeks spec	73.	267.963	{21., 604.}	292.593	0.703622	newly 3 weeks cumul	110.	462.574	{38., 994.}	343.822	0.721781	newly 5 weeks spec	151.	752.315	{80., 1328.}	418.163	0.879793	newly 5 weeks cumul	352.	1698.61	{139., 3328.}	403.667	0.851065	newly 65 weeks cumul	4475.	7688.76	{5510., 9196.}	86.7995	0.561243
48	newly 3 weeks spec	120.	171.583	{0., 350.}	93.6806	0.755195	newly 3 weeks cumul	221.	540.583	{9., 1049.}	185.784	0.616473	newly 5 weeks spec	260.	130.5	{0., 353.}	66.3462	0.988472	newly 5 weeks cumul	652.	832.917	{9., 1519.}	79.205	0.538265	newly 62 weeks cumul	3589.	1245.75	{9., 2803.}	65.2898	0.821197
49	newly 3 weeks spec	81.	361.115	{140., 560.}	345.821	0.607929	newly 3 weeks cumul	266.	1269.12	{716., 1714.}	377.111	0.658891	newly 5 weeks spec	116.	222.154	{57., 503.}	113.594	0.309221	newly 5 weeks cumul	474.	1770.42	{821., 2404.}	273.507	0.544476	newly 58 weeks cumul	3702.	2292.85	{917., 3855.}	40.367	0.260981
50	newly 3 weeks spec	137.	210.813	{83., 472.}	81.3412	0.2907	newly 3 weeks cumul	367.	826.875	{471., 1428.}	133.719	0.345968	newly 5 weeks spec	235.	131.938	{16., 534.}	76.0372	0.701508	newly 5 weeks cumul	808.	1127.38	{551., 2076.}	64.4338	0.240559	newly 58 weeks cumul	2666.	1443.81	{555., 3363.}	55.2115	0.417392
